# Evaluation of Positional Variability in Surface Roughness in Orthodontic Mini‐Implants Using Confocal Microscopy: A Multi‐Manufacturer Comparative Study

**DOI:** 10.1002/cre2.70497

**Published:** 2026-10-29

**Authors:** Alessandro Antonelli, Till Augustinowitz, Giulia Brunello, Kathrin Becker

**Affiliations:** ^1^ Department of Orthodontics and Dentofacial Orthopaedics Charité – Universitätsmedizin Berlin Berlin Germany; ^2^ Health Sciences Department, School of Dentistry Magna Graecia University of Catanzaro Catanzaro Italy; ^3^ Department of Oral Surgery University Hospital Düsseldorf Düsseldorf Germany

**Keywords:** confocal laser scanning microscopy, orthodontic mini‐implants, surface roughness

## Abstract

**Objective:**

To compare the surface roughness of orthodontic mini‐implants (OMIs) from seven manufacturers at standardized positions, since positional variability in OMI topography has not been systematically investigated but may influence healing and stability.

**Materials and Methods:**

Seven OMI systems (*n* = 5 per system, total *n* = 35) were analyzed using 3D confocal laser scanning microscopy. Four locations per OMI were assessed (apex, mid, top, neck). Roughness parameters included Sa and Sz (µm), Sdr (%), Str (dimensionless), and Spc (1/mm). Linear mixed‐effects models were fitted for each parameter with Manufacturer and Location as fixed effects and Implant ID as a random intercept. Fixed effects were evaluated by Type III ANOVA; estimated marginal means (EMMs, 95% CIs) and Tukey‐adjusted pairwise contrasts summarized differences.

**Results:**

Manufacturer, location, and their interaction were significant for all parameters (*p* < 0.0001). Dentaurum consistently showed the highest mean roughness values, especially at the neck, with an overall average value for Sa ≈ 0.81 µm compared to 0.20–0.39 µm for others, Sz ≈ 9.5 µm (others 2–3 µm), and Sdr ≈ 6%–7% (others ≤ 2%). The mean Spc at the neck reached ≈65,000 1/mm for Dentaurum, while OBS showed markedly lower mean values (< 10,000 1/mm). Ortholox Plus (TigerDental) showed intermediate increases at the neck, while OBS presented the smoothest surfaces, particularly at the neck. Str indicated manufacturer‐specific isotropy: American Orthodontics and Forestadent demonstrated higher Str mean values (≈0.7–1.0) at the top and neck, whereas AbsoAnchor, Dentaurum, DualTop, OBS, and Ortholox Plus showed lower Str.

**Conclusions:**

OMI surface roughness is strongly manufacturer‐ and position‐dependent, with the neck region amplifying inter‐system differences. Selecting OMIs from the market or customizing their design based on anatomical considerations and applied forces may optimize osseointegration and peri‐implant soft tissue health. Adjusting roughness profiles may help reduce implant migration. While long smooth necks might be advantageous in cases of extended transmucosal path.

## 1 Introduction

Orthodontic mini‐implants (OMIs) became indispensable tools in contemporary orthodontics to provide reliable skeletal anchorage for complex tooth movements and enable the application of orthopedic forces (Becker et al. 2018; Nienkemper et al. 2013). Since their introduction in the late 1990s by Kanomi et al. OMIs revolutionized orthodontics by offering a minimally invasive and cost‐effective solution for anchorage reinforcement (Kanomi 1997). After their inception, numerous variants in screw design and surface morphology have been developed and marketed by various manufacturers (Baumgaertel et al. 2008).

Most OMIs are fabricated from titanium alloy, specifically Ti‐6Al‐4V (Titanium Grade 5), known for their biocompatibility. Additionally, this material ensures resistance to corrosion, favorable tissue response, and mechanical stability essential for orthodontic loading (Alves et al. 2016). Nonetheless, success rates of OMIs vary significantly, influenced primarily by multiple clinical factors, but also biological factors where surface characteristics play a crucial role (Chaddad et al. 2008; Topouzelis and Tsaousoglou 2012).

It is known from implant dentistry that surface roughness, one of the most critical aspects of implant surface properties, governs the initial mechanical interlocking of the implant within bone and the biological response elicited at the bone‐implant and soft tissue‐implant interfaces. Surface roughness encompasses the microscopic irregularities and topographical features of the OMI surface that mediate the degree of mechanical retention and osseointegration (Parithimarkalaignan and Padmanabhan 2013). Previous studies have demonstrated that moderately roughened surfaces enhance greater bone‐implant contact, thereby promoting primary stability, which is essential for immediate or early loading during orthodontic treatment (Baró et al. 1986).

In contrast, the OMI neck, which transverses the oral mucosa, benefits from a smoother surface to reduce plaque and biofilm accumulation and minimize soft tissue inflammation (Ferreira et al. 2015; Guo et al. 2021).

In contrast to the extensive surface treatments used in conventional dental implants, such as sandblasting, acid etching, and anodization, OMIs predominantly undergo machining processes, with primary stability largely relying on macroretention (van den Braak et al. 2024). Despite this, comparative studies evaluating surface roughness across diverse OMI types remain limited, because only the surface of singular OMI types has been investigated so far (Eliades et al. 2009). Furthermore, no differentiated analysis of the positional variability of OMIs has been conducted to date, even though the neck region of OMIs exhibits distinctly different biological requirements compared to their intraosseous portions. One possible reason for the limited investigation of positional variability is the assumption that mini‐implant surfaces are sufficiently homogeneous for measurements from selected areas to represent overall surface roughness. However, the neck region of OMIs exhibits distinctly different biological requirements compared to their intraosseous portions.

The advent of advanced surface metrology techniques, including confocal laser scanning microscopy, has greatly enhanced the ability to characterize surface roughness non‐destructively and with nanometer‐scale resolution. This multiparametric approach facilitates detailed quantification of roughness features without damaging the sample, making it exceptionally suited for analyzing the surface of OMIs (García et al. 2018).

Moreover, 3D confocal laser scanning microscopy has facilitated discerning roughness differences in implant dentistry at various implant regions, which is essential since the thread, neck, and head regions undergo different mechanical and biological interactions (Beger et al. 2018).

Current literature emphasizes that surface roughness parameters directly correlate with implant stability and biological integration, but the extent of positional variability in terms of OMIs remains underexplored.

Furthermore, clinically relevant investigations that link surface roughness variability to biomechanical stability and biological performance are urgently needed to select OMIs with tailored surface properties. Despite this, the positional variability in surface topography among commercially available OMIs remains under‐investigated. To date, no systematic analysis has addressed how roughness varies along different OMI regions, although such positional heterogeneity could influence both osseointegration and transmucosal tissue health. Understanding these patterns may help explain differences in clinical performance and guide the development of optimized implant designs.

Therefore, the aim of this study was to analyze the positional variability in surface roughness of seven different types of OMIs employing innovative high‐resolution 3D confocal laser scanning microscopy. By precisely quantifying key surface parameters at standardized locations on each OMI, this research aims to reveal differences in surface architecture between manufacturers and within the OMIs themselves, providing insights into OMI design variability and potential clinical performance.

## 2 Materials and Methods

This study was reported according to the modified CONSORT guidelines of Faggion (2012).

### 2.1 Sample Overview

A total of seven different types of OMIs by different manufacturers were analyzed in this study. Five samples per manufacturer were characterized, based on a convenience sampling approach, as no comparable data were available at the time of study planning to guide a prospective sample size calculation.

All analyzed OMIs were composed of a titanium‐aluminum‐vanadium alloy (Ti‐6Al‐4V; Grade 5 titanium) and were evaluated directly out of their original packaging without prior clinical insertion or handling.

The first examined sample was the AbsoAnchor OMI (Dentos Inc., Daegu, South Korea), featuring a diameter of 2 mm and a length of 8 mm with a small head (SH) design.

The American Orthodontics OMI (Sheboygan, Wisconsin, USA) had a diameter of 1.5 mm and a length of 11.2 mm with a bracket‐type head.

The Dentaurum tomas®‐pin SD 10 (Ispringen, Germany) possessed a diameter of 1.6 mm and a length of 10 mm, characterized by a 22 cross‐slot head.

The Dual‐Top^TM^ G2 OMI (Jeil Medical, Seoul, South Korea) also features a bracket‐type head with an additional hole and has a diameter of 1.6 mm and a length of 10 mm.

The OBS OMI (BOMEI Co. Ltd., Taoyuan City, Taiwan) presented a diameter of 2 mm and a length of 12 mm, incorporating two design features: a smooth mushroom head designed for elastic chain retention and additional 4‐way rectangular holes for wire mechanics such as lever arms.

The OrthoEasy® Pin (Forestadent, Pforzheim, Germany) measured 1.7 mm in diameter and 6 mm in length; the head incorporates a cross‐slot bracket design. The transgingival neck height is 1.5 mm with a tapered transition to the thread.

The analyzed Plus OMI (TigerDental, Freiburg, Germany) measured 2 mm in diameter and 10 mm in length. It featured a head equipped with a fixation system utilizing a cover screw to facilitate attachment of various appliances (Table 1; Figure 1).

**Table 1:** Summary of the OMI description.

OMI type and manufacturer	Ref	Diameter (mm)	Length (mm)	Head design
AbsoAnchor (Dentos Inc., Daegu, South Korea)	ϒ‐SH2018‐08	2	8	Small head (SH)
American Orthodontics (Sheboygan, Wisconsin, USA)	68.99.30 A	1.5	11.2	Bracket head
tomas®‐pin SD (Dentaurum, Ispringen, Germany)	302‐110‐00	1.6	10	Bracket head
Dual‐Top^TM^ (Jeil Medical, Seoul, South Korea)	S16‐G2‐010N	1.6	10	Bracket head with additional hole
OBS (BOMEI Co. Ltd., Taoyuan City, Taiwan)	BD‐2107‐0	2	12	Mushroom head and 4‐way rectangular holes
OrthoEasy® Pin (Forestadent, Pforzheim, Germany)	1101A2306	1.7	6	Bracket head
Ortholox Plus (TigerDental, Freiburg, Germany)	20 OLX 010	2	10	Interchangeable abutment system

**Figure 1:**
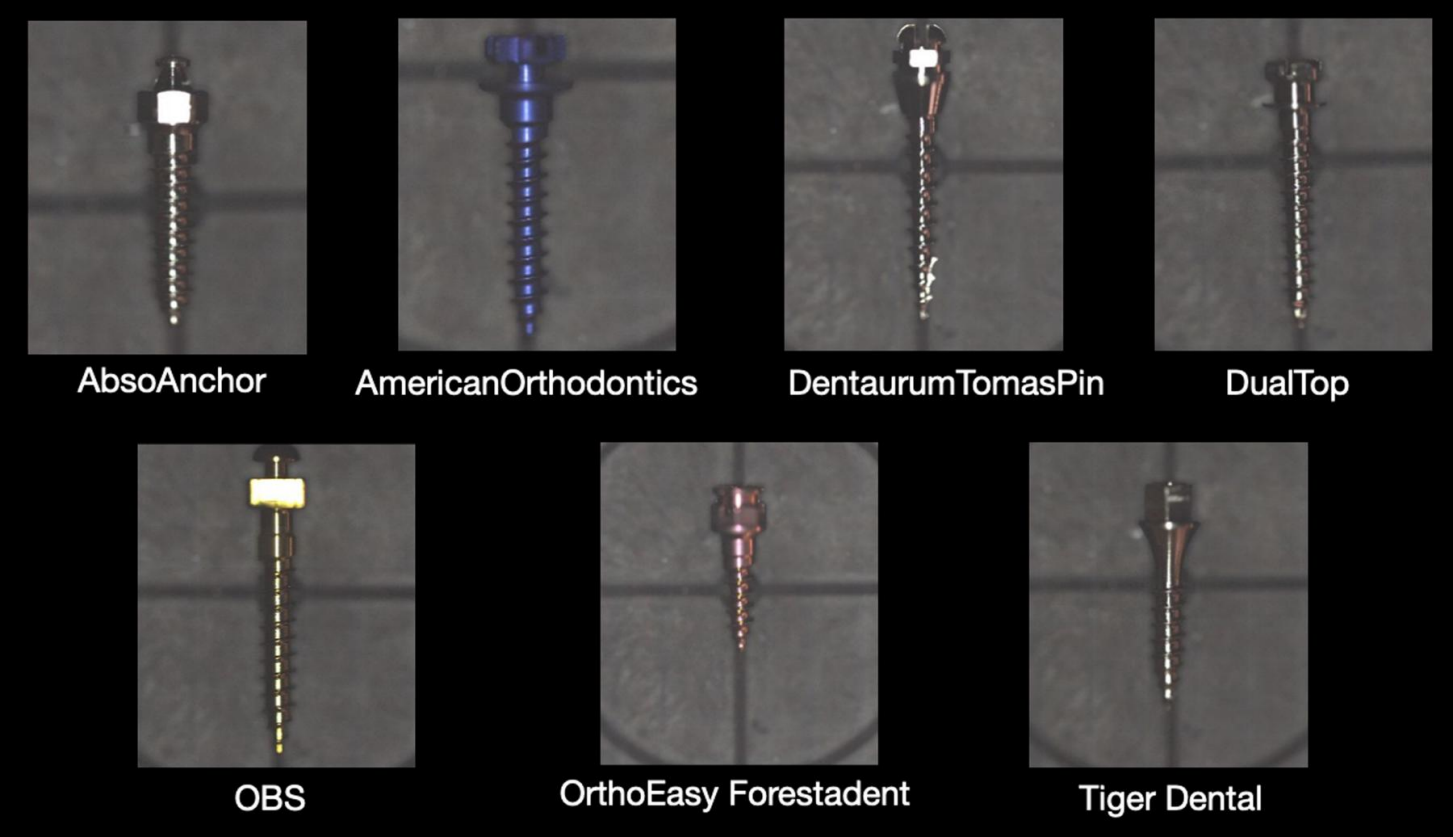
Overview of the different types of OMIs that were analyzed.

### 2.2 Surface Analysis

The surface characterization was performed on five distinct samples of each type of OMI using a 3D Confocal Laser Scanning Microscope (VK‐X 3000 Viewer Analysis, VK‐X3000, KEYENCE, Osaka, Japan). OMI images were first acquired at 5× magnification (Plan 5× WD22.5 lens) using white light illumination. For each specimen, four selected regions of interest (ROI; 208.29 μm × 277.81 μm) were further examined at 20× magnification (Plan 20× WD0.54 lens) under laser confocal mode. Consistent acquisition settings were maintained by controlling the light source power at level 30 and maximizing the field of view (Figure 2).

**Figure 2:**
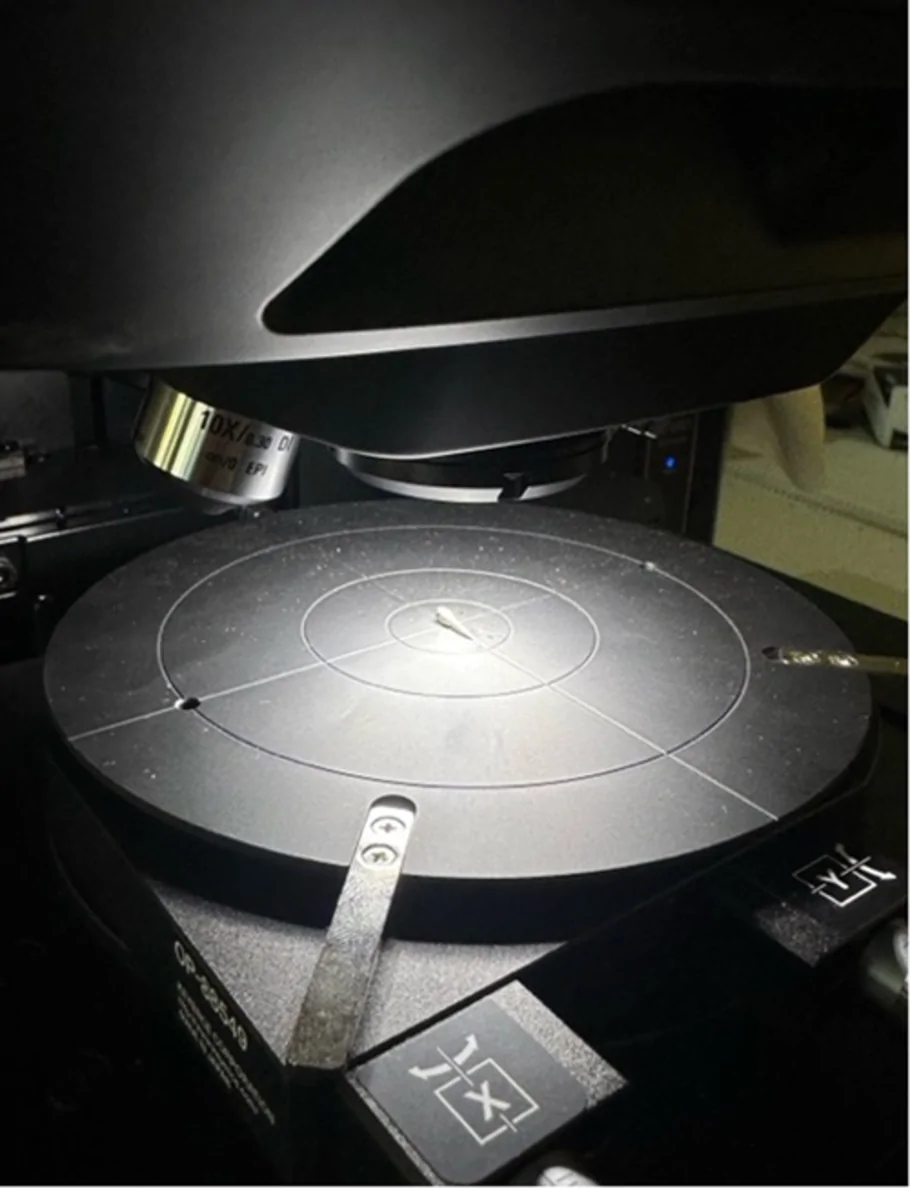
3D confocal laser scanning microscope (VK‐X3000, KEYENCE, Osaka, Japan) performing OMI analysis.

Surface characterization was conducted at four standardized locations per OMI. Three analysis points were selected along the screw thread: (1) the distal end near the OMI apex (apex), (2) a valley located centrally on the fixture (mid), and (3) the proximal area adjacent to the bone‐contacting neck region (top).

The fourth point was located on the OMI neck (4).

By selecting these specific points, the neck region—with its distinct functional requirements—can be evaluated independently. Furthermore, the division of the OMI thread into three measurement points allows detailed assessment of positional variability along the screw, enabling the identification of localized differences in thread geometry and surface characteristics (Figure 3).

**Figure 3:**
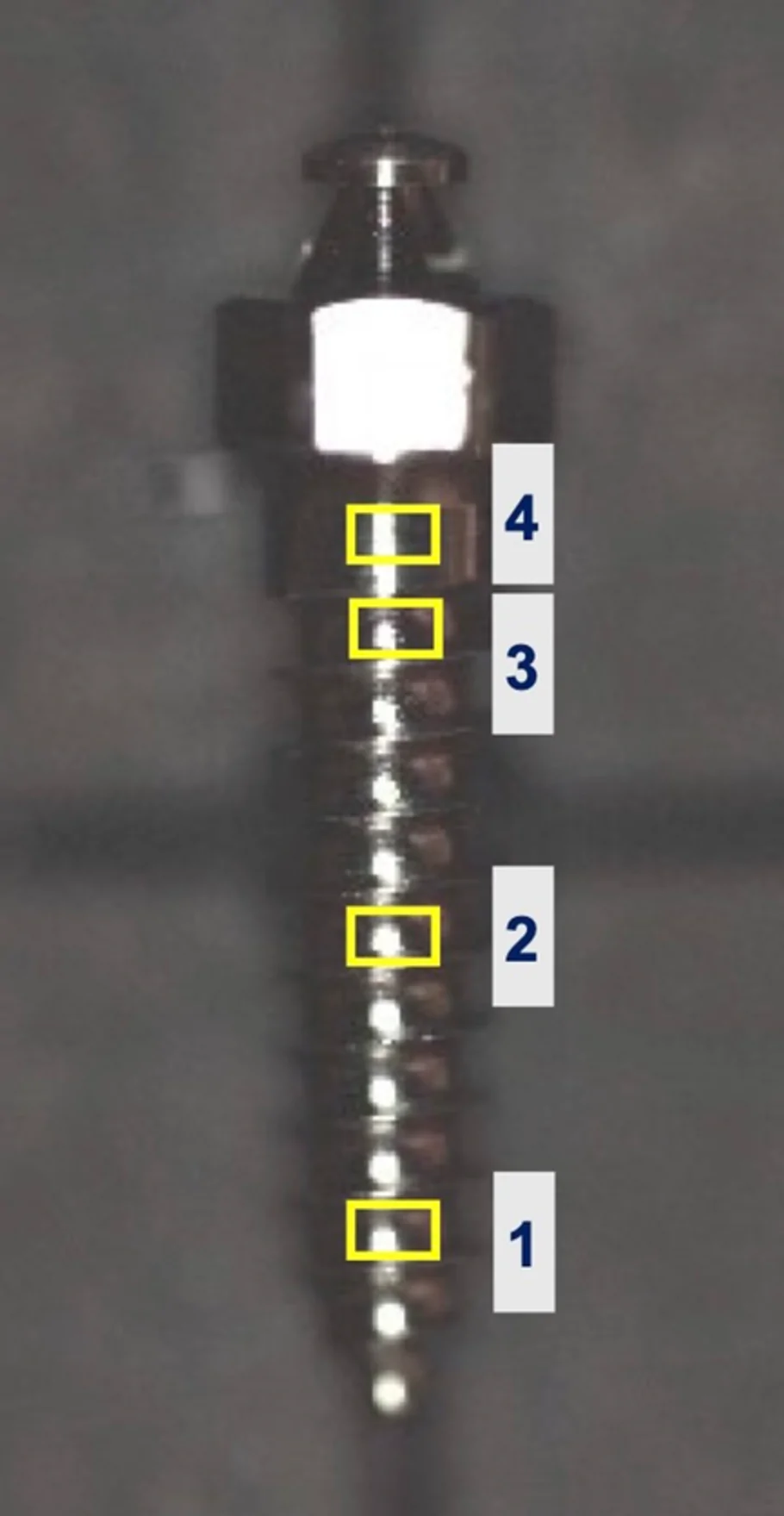
Visualization of the four measurement points using the example of a Dentaurum tomas®‐OMI.

The acquired scans were processed with the VK‐X3000 MultiFileAnalyzer software (KEYENCE), performing “Surface shape correction ‐ Cylinder.” A central subarea of 200 μm × 200 μm from each scan was then analyzed for surface roughness. Roughness parameters were quantified according to ISO 25178 guidelines with an L‐filter of 0.25 (Figure 4).

**Figure 4:**
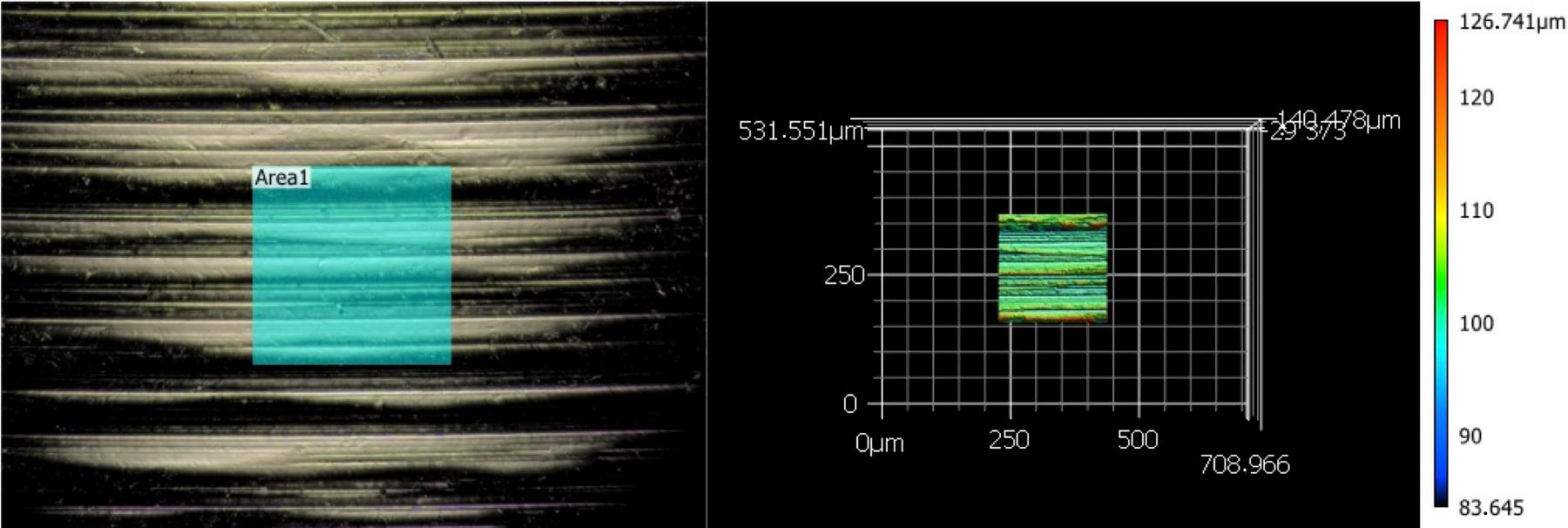
Representative image of a laser‐based optical surface roughness measurement on an OMI (Dentaurum tomas®). Representative three‐dimensional renderings of the analyzed digital optical impression (left) are shown alongside the corresponding surface height map (right). Measurements were performed on a standardized area of interest (200 μm × 200 μm; Area 1), highlighted on the raw optical images. Color‐coded map indicates surface height variations (in μm), generated using the MultiFileAnalyzer software (KEYENCE, Osaka, Japan).

### 2.3 The Surface Analysis Was Determined Using the Following Parameters

- Sa (Arithmetical Mean Height), indicating the average absolute deviation of surface heights from the mean plane across a defined area; higher values correlate with increased roughness
- Sz (Maximum Height), combining the highest peak and deepest valley heights within the area, represents vertical surface extremes. Both Sa and Sz are reported in micrometers (µm) (Rosentritt et al. 2024).
- Str (Texture Aspect Ratio), a dimensionless ratio ranging from 0 to 1, describes the isotropy of the surface texture, with values closer to 1 indicating isotropic texture and lower values reflecting anisotropic feature.
- Sdr (Developed Interfacial Area Ratio), expressed as a percentage (%), quantifies the expanded surface area due to texture relative to the projected planar area; a higher Sdr value reflects a more complex, three‐dimensional surface topography, which can considerably influence biological and mechanical interactions (Keyence 2024).
- Spc (Arithmetic Mean Peak Curvature) measures the average curvature of surface peaks, characterizing peak sharpness or bluntness, which can influence biological adhesion and material interactions; smaller values indicate more rounded or smooth peak contacts, while larger values correspond to sharper, more pointed peaks. Therefore, this parameter is important for characterizing surface interactions such as biological adhesion. Spc is expressed in units of inverse length (1/mm) (Kováč et al. 2021).

### 2.4 Statistical Analysis

Surface roughness values were analyzed using a linear mixed‐effects model to account for the dependency of repeated measurements at different locations within the same OMI. The model included manufacturer and measurement location (apex, mid, neck, top) as fixed effects, as well as their interaction term. Implant ID was included as a random intercept to account for within‐implant correlation. Models were fit using restricted maximum likelihood estimation (REML).

Model assumptions were verified using residual and quantile–quantile plots. Residual plots were also inspected for influential or extreme values and all measurements were retained in the analysis. Type III analysis of variance (ANOVA) was performed to test main and interaction effects. Estimated marginal means (EMMs) with 95% confidence intervals (CIs) were computed, and post hoc pairwise comparisons were conducted with Tukey's adjustment for multiple testing. Effect sizes were quantified using marginal and conditional *R* ^2^ values for the mixed‐effects models. For post hoc comparisons, standardized mean differences (d) were calculated by dividing estimated marginal mean differences by the model residual standard deviation, allowing assessment of the magnitude of location‐ and manufacturer‐specific effects independent of sample size. In addition, a post hoc sensitivity analysis was performed for the Sa outcome to support interpretation of the manufacturer‐specific pairwise contrasts within each measurement location. Minimum detectable differences (at 80% power) were estimated from group sizes and converted to µm using the model's residual standard deviation. Since actual comparisons used Tukey adjustment, these thresholds serve as approximate, not exact, benchmarks for detectability.

All analyses were performed in R (version 2025, R Foundation for Statistical Computing, Vienna, Austria) using the lme4, car, and emmeans packages. Statistical significance was set at *p* < 0.05.

## 3 Results

A summary of the surface roughness findings, including the mean and standard deviation (SD) for each implant type and location, is presented in Table 2.

**Table 2:** Surface roughness. Data are expressed as mean (SD).

OMI type	Location	Sa (µm)	Sz (µm)	Sdr (%)	Str	Spc (1/mm)
AbsoAnchor	Apex	0.409 (0.027)	20.078 (3.521)	0.501 (0.051)	0.117 (0.007)	2327 (161)
	Mid	0.426 (0.026)	23.446 (2.572)	0.550 (0.056)	0.120 (0.018)	2421 (148)
	Top	0.475 (0.047)	22.014 (1.112)	0.668 (0.144)	0.125 (0.014)	2807 (346)
	Neck	0.246 (0.049)	4.825 (0.605)	0.134 (0.036)	0.041 (0.045)	1221 (107)
American Orthodontics	Apex	0.280 (0.067)	15.939 (8.300)	0.358 (0.140)	0.651 (0.128)	2066 (555)
	Mid	0.288 (0.018)	11.464 (6.559)	0.364 (0.079)	0.763 (0.098)	2160 (299)
	Top	0.311 (0.055)	6.556 (1.386)	0.289 (0.087)	0.366 (0.416)	1880 (372)
	Neck	0.276 (0.053)	5.391 (0.484)	0.205 (0.086)	0.322 (0.388)	1553 (346)
tomas®‐pin (Dentaurum)	Apex	0.224 (0.020)	18.565 (2.042)	0.182 (0.037)	0.052 (0.030)	1489 (180)
	Mid	0.202 (0.019)	19.889 (1.637)	0.176 (0.024)	0.033 (0.019)	1406 (122)
	Top	0.144 (0.047)	8.226 (3.823)	0.073 (0.083)	0.021 (0.006)	861 (605)
	Neck	2.673 (0.469)	66.145 (49.467)	5.065 (1.169)	0.025 (0.010)	10495 (1326)
Dual‐Top^TM^	Apex	0.280 (0.055)	12.787 (2.039)	0.255 (0.097)	0.104 (0.016)	1766 (255)
	Mid	0.192 (0.021)	9.988 (1.481)	0.131 (0.032)	0.093 (0.018)	1314 (133)
	Top	0.198 (0.017)	11.626 (2.749)	0.156 (0.055)	0.102 (0.106)	1337 (174)
	Neck	0.137 (0.020)	3.924 (0.503)	0.042 (0.017)	0.015 (0.002)	684 (123)
OBS	Apex	0.280 (0.159)	18.497 (6.195)	0.366 (0.378)	0.026 (0.006)	2093 (1098)
	Mid	0.276 (0.119)	14.914 (5.873)	0.277 (0.258)	0.020 (0.005)	1588 (745)
	Top	0.258 (0.087)	17.193 (6.704)	0.244 (0.179)	0.021 (0.005)	1669 (380)
	Neck	0.052 (0.021)	2.485 (0.424)	0.005 (0.004)	0.132 (0.228)	250 (151)
OrthoEasy® (Forestadent)	Apex	0.275 (0.031)	16.923 (3.151)	0.335 (0.091)	0.461 (0.166)	1964 (243)
	Mid	0.263 (0.017)	13.999 (3.223)	0.299 (0.031)	0.633 (0.064)	1857 (116)
	Top	0.279 (0.037)	8.577 (3.431)	0.282 (0.193)	0.224 (0.296)	1611 (740)
	Neck	0.203 (0.032)	4.130 (1.411)	0.141 (0.072)	0.874 (0.027)	1169 (507)
Ortholox Plus (TigerDental)	Apex	0.200 (0.023)	8.153 (1.481)	0.136 (0.060)	0.046 (0.010)	1196 (299)
	Mid	0.187 (0.020)	9.401 (1.261)	0.151 (0.038)	0.060 (0.021)	1203 (164)
	Top	0.212 (0.030)	9.006 (1.311)	0.150 (0.073)	0.072 (0.011)	1249 (372)
	Neck	0.744 (0.166)	17.704 (7.801)	1.259 (0.651)	0.078 (0.040)	4257 (1422)

The surface roughness parameters Sa, Sz, Sdr, Str, and Spc were analyzed using linear mixed‐effects models with manufacturer and measurement location as fixed factors and implant ID as a random intercept. For all parameters, Type III ANOVA demonstrated significant main effects of manufacturer and location, as well as highly significant interaction effects (all *p* < 0.0001), confirming that surface topography differed systematically both between OMI systems and across OMI positions.

### 3.1 Arithmetical Mean Height (Sa)

The linear mixed‐effects model revealed significant effects of Manufacturer (*F* = 57.4), Measurement Location (*F* = 103.7), and their interaction (*F* = 115.7; all *p* < 0.0001). The mixed‐effects model demonstrated a very high explanatory power, with a marginal *R* ^2^ of 0.95 and a conditional *R* ^2^ of 0.96, indicating that manufacturer, measurement location, and their interaction explained nearly all variance in Sa values, with minimal additional variance attributable to implant‐level random effects. Estimated marginal means indicated that Dentaurum OMIs exhibited substantially higher Sa values (0.81 µm, 95% CI: 0.76–0.87) compared to all other systems (range: 0.20–0.39 µm, *p* < 0.0001). Notably, the 95% confidence intervals for Dentaurum did not overlap with those of any other manufacturer across measurement locations, indicating a difference in mean surface roughness. In contrast, confidence intervals among the remaining systems partially overlapped, suggesting more comparable Sa profiles between these manufacturers. The significant Manufacturer × Location interaction demonstrated that roughness differences were not uniform across OMI positions and varied substantially between manufacturers (Figure 5). Within manufacturers, significant location‐specific differences were observed for Dentaurum (extreme increase at the neck), Ortholox Plus (increase at the neck), OBS (reduction at the neck), and AbsoAnchor (lower values at the neck). Representative images of all investigated OMIs are provided in Figure 6 and Figures S1–S3.

**Figure 5:**
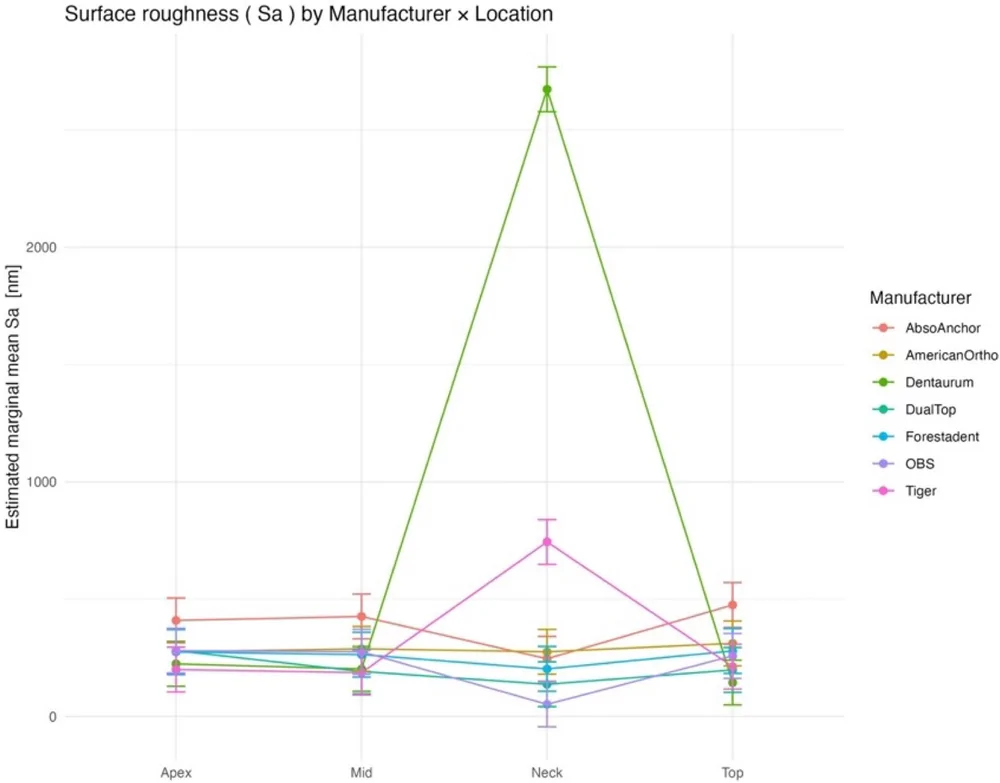
Estimated marginal means (±95% CI) of Sa values (µm) for each manufacturer across measurement locations. Results derived from linear mixed‐effects modeling.

**Figure 6:**
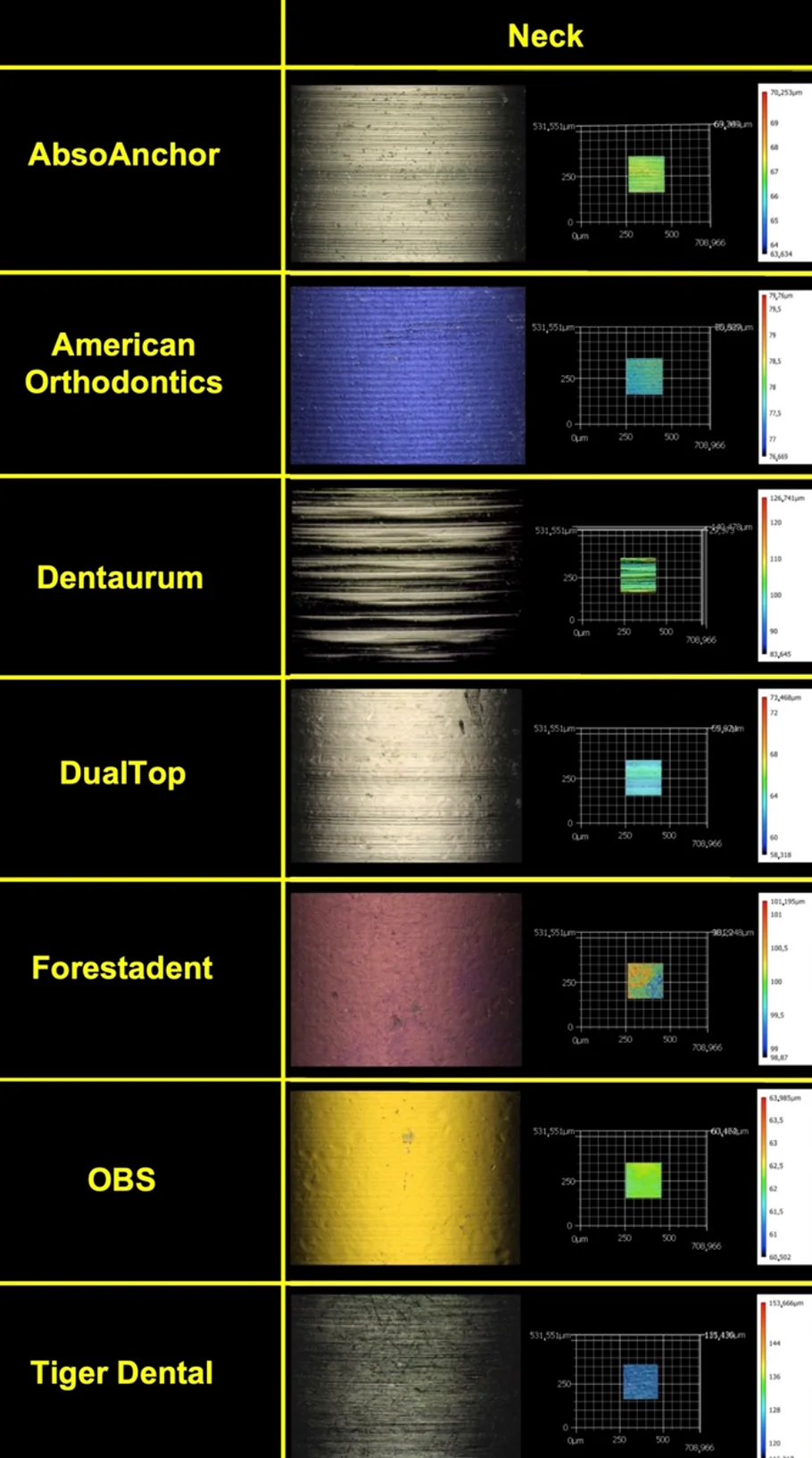
Illustration of laser‐based optical surface roughness measurements at the neck for all seven manufacturers with related rendering of the analyzed digital optical impression (200 μm × 200 μm), created with MultiFileAnalyzer software (KEYENCE, Osaka, Japan).

Post hoc pairwise comparisons of estimated marginal means revealed marked differences between specific manufacturer–location combinations, with standardized mean differences ranging from 1.6 to 1.8. These findings indicate substantial differences in surface roughness at distinct implant regions and underscore the strong manufacturer‐specific modulation of location‐dependent roughness patterns.

To facilitate interpretation of these Tukey‐adjusted comparisons, an additional post hoc sensitivity analysis was conducted for Sa. This analysis provided approximate benchmarks for the minimum between‐manufacturer differences in Sa that could be detected with 80% power within each location. The sensitivity analysis showed that the larger between‐manufacturer separations observed in the neck region more often exceeded the detectability thresholds, whereas smaller contrasts at apex, mid, and Top less frequently did so. The detailed results of the post hoc sensitivity analysis for Sa are provided in Table S1.

### 3.2 Maximum Height (Sz)

The linear mixed‐effects model revealed highly significant effects of Manufacturer, Measurement Location, and their interaction (all *p* < 0.0001). The model showed a moderate‐to‐high explanatory power, with a marginal *R* ^2^ of 0.57 and a conditional *R* ^2^ of 0.58, indicating that the majority of variance in Sz values was explained by fixed effects, while implant‐level random effects contributed only minimally. Estimated marginal means demonstrated that Dentaurum OMIs displayed extreme Sz values (≈9.5 µm at the neck), far exceeding those of all other systems (≈2–3 µm), with all contrasts highly significant (*p* < 0.0001). Ortholox Plus exhibited intermediate values with a pronounced increase at the neck (≈3 µm).

Analysis of the maximum height parameter (Sz), reflecting vertical surface extremes, further demonstrated that the neck region exhibited the lowest Sz values in nearly all OMI types, except for Dentaurum and Ortholox Plus. Within the group of OMIs displaying the lowest mean values, data dispersion was minimal, suggesting consistency across samples. This observation was supported by comparatively narrow 95% confidence intervals for these systems, indicating low variability and stable Sz values across samples. In contrast, Dentaurum OMIs exhibited markedly wider confidence intervals at the neck, reflecting substantial heterogeneity in vertical surface extremes at this location. OBS OMIs yielded the lowest Sz values within this cohort. Post hoc pairwise comparisons revealed large to extremely large standardized differences between specific manufacturer–location combinations, with standardized mean differences ranging from approximately *d* = 1.5 to *d* > 5.0, underscoring substantial and manufacturer‐specific modulation of vertical surface extremes across implant regions. Collectively, these results confirm that Sz varied markedly by position, with extreme peaks at the neck for Dentaurum and, to a lesser degree, for Ortholox Plus, while the other systems showed relatively stable or even reduced values across positions (Figure 7).

**Figure 7:**
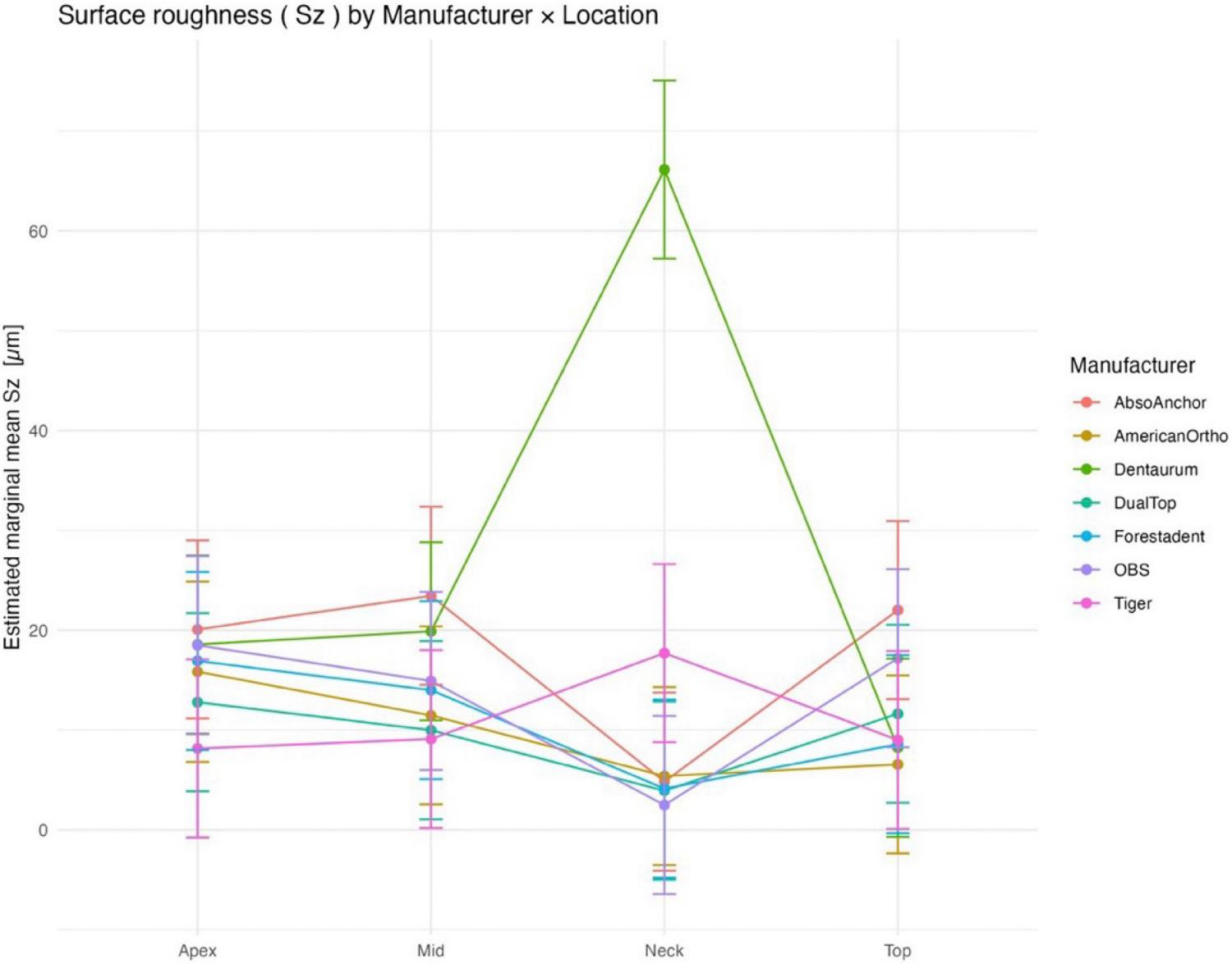
Estimated marginal means (±95% CI) of Sz values (µm) for each manufacturer across measurement locations.

### 3.3 Developed Interfacial Area Ratio (Sdr)

The analysis of Sdr showed significant main and interaction effects (all *p* < 0.0001). The model demonstrated a high explanatory power, with a marginal *R* ^2^ of 0.92 and a conditional *R* ^2^ of 0.92, indicating that the vast majority of variance in Sdr values was explained by fixed effects, with only a small additional proportion attributable to implant‐level random effects. Dentaurum OMIs exhibited the highest Sdr values, reaching ≈6%–7% at the neck, compared with ≤ 2% for all other systems, with contrasts highly significant (*p* < 0.0001). The absence of overlap between the 95% confidence intervals of Dentaurum and those of the remaining systems at the neck further underscores the pronounced difference in surface area development at this location. Ortholox Plus demonstrated intermediate Sdr values (≈2%–3%).

In the majority of systems (AbsoAnchor, AmericanOrtho, DualTop, Forestadent, OBS), the lowest mean Sdr values were consistently observed at the neck, suggesting reduced surface area development in this region. In contrast, Dentaurum presented a pronounced exception, with neck means that were distinctly higher than those of other positions and manufacturers, reflecting enhanced topographical complexity. OBS OMIs exhibited the lowest mean Sdr values at the neck, supporting the presence of a comparatively smooth transmucosal region. Additionally, AmericanOrtho and Forestadent displayed elevated Sdr values at the top, suggesting increased surface complexity toward the exposed coronal regions (Figure 8).

**Figure 8:**
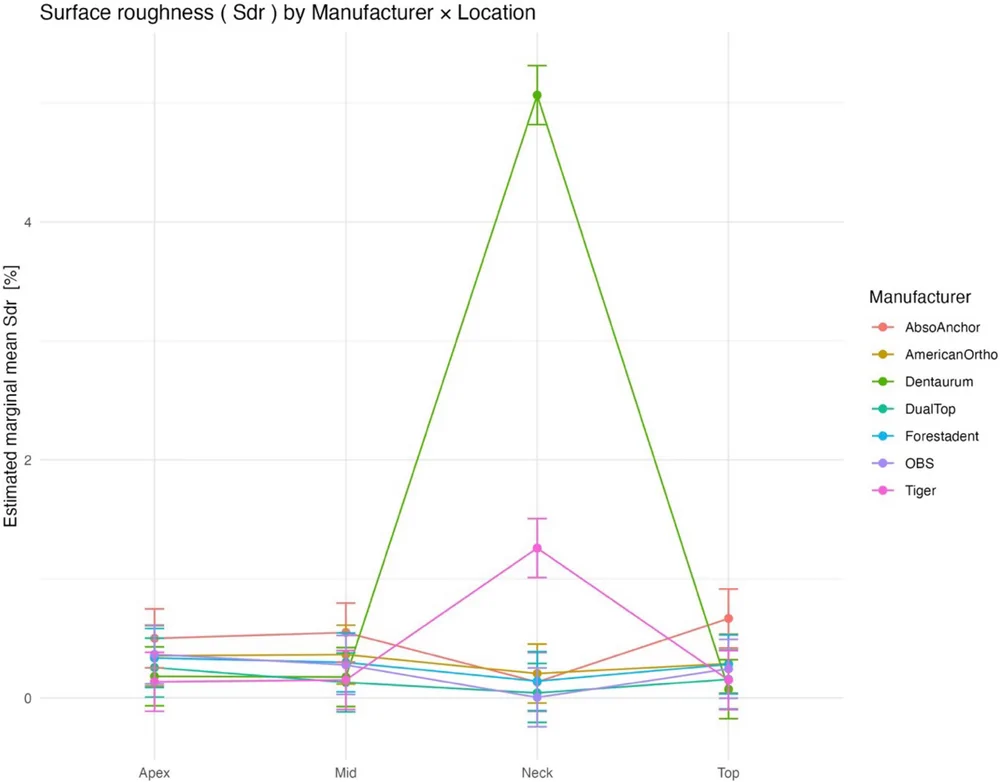
Estimated marginal means (±95% CI) of Sdr values (%) for each manufacturer across measurement locations.

Post hoc pairwise comparisons revealed large standardized differences between specific manufacturer–location combinations, with standardized mean differences reaching approximately *d* ≈ 1.3–1.6, underscoring substantial and manufacturer‐specific modulation of surface area development across implant regions.

### 3.4 Texture Aspect Ratio (Str)

For Str, the mixed‐effects model indicated significant differences between manufacturers and locations, as well as a significant interaction effect (all *p* < 0.0001). The model demonstrated a moderate explanatory power, with a marginal *R* ^2^ of 0.63 and a conditional *R* ^2^ of 0.65, indicating that a substantial proportion of variability in Str values was explained by fixed effects, while implant‐level random effects contributed only a small additional proportion of variance. Estimated marginal means showed pronounced differences in surface isotropy between manufacturers, with Str values ranging from approximately 0.05 to 1.0.

AbsoAnchor, Dentaurum, DualTop, OBS, and Ortholox Plus generally showed low Str means across positions, indicating predominantly anisotropic surfaces. By contrast, AmericanOrtho and Forestadent demonstrated substantially higher Str values, often exceeding 0.7 at the neck and top, consistent with isotropic surface characteristics.

Within‐system variability differed considerably: AmericanOrtho OMIs displayed the broadest variation in Str, particularly at the neck and top, whereas Forestadent OMIs showed consistently high and uniform Str values at the mid and top, suggesting a more stable isotropic surface texture (Figure 9). This pattern was accompanied by narrow confidence intervals, indicating a stable and reproducible isotropic surface texture, whereas AmericanOrtho displayed wider confidence intervals at the neck and top, reflecting greater within‐system variability.

**Figure 9:**
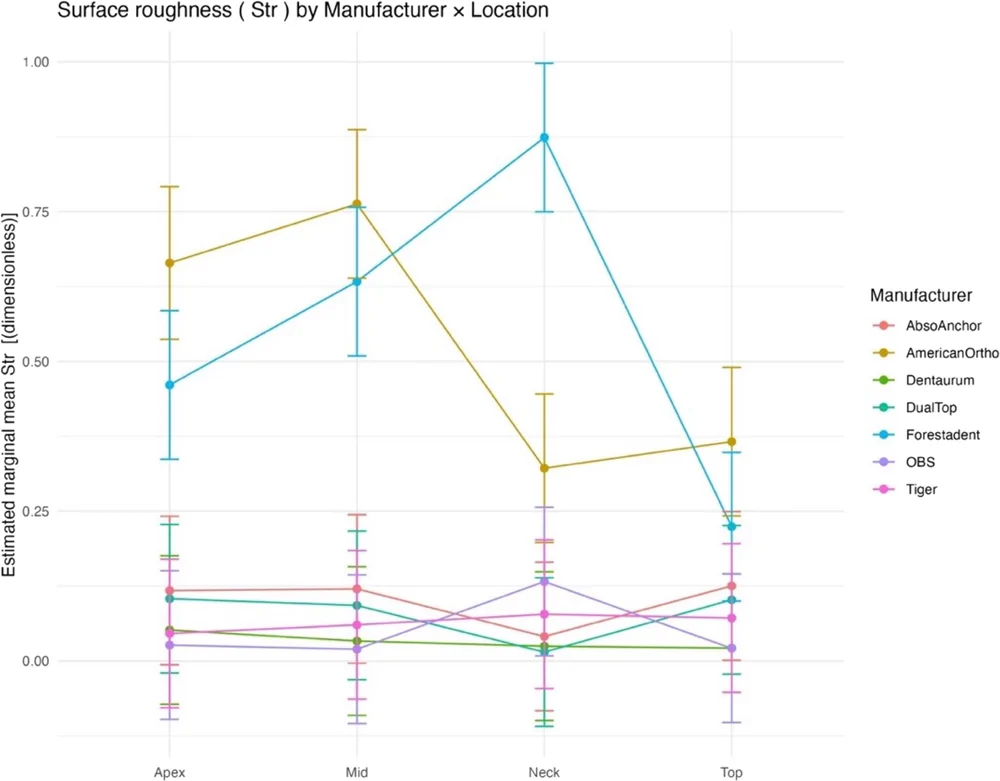
Estimated marginal means (±95% CI) of Str values (dimensionless) for each manufacturer across measurement locations.

Post hoc pairwise comparisons revealed moderate to large standardized differences between specific manufacturer–location combinations, with standardized mean differences reaching approximately *d* ≈ 0.8–1.4, underscoring meaningful manufacturer‐specific modulation of surface texture isotropy across implant regions.

### 3.5 Peak Density (Spc)

The linear mixed‐effects model revealed significant effects of Manufacturer, Measurement Location, and their interaction (all *p* < 0.0001). The model showed a high explanatory power, with a marginal *R* ^2^ of 0.89 and a conditional *R* ^2^ of 0.90, indicating that most of the variability in Spc values was explained by fixed effects, with only a small additional contribution from implant‐level random effects. Estimated marginal means indicated that Dentaurum OMIs displayed dramatically higher peak densities (≈65,000 1/mm at the neck) compared with all other systems (≈15,000–25,000 1/mm), with all contrasts highly significant (*p* < 0.0001). OBS OMIs consistently showed the lowest Spc values (< 10,000 1/mm), particularly at the neck, reflecting a smoother surface with fewer peaks.

A consistent pattern was observed across most OMI types: the neck region exhibited the lowest mean Spc values, except in Dentaurum and Ortholox Plus, where peak density increased significantly at this location. For systems with reduced Spc at the neck, confidence intervals were comparatively narrow, indicating low variability. In contrast, Dentaurum OMIs combined the highest means with the broadest dispersion, especially at the neck, reflecting heterogeneous peak distribution (Figure 10). This was reflected in wide 95% confidence intervals at the neck region, indicating substantial variability in peak density within this system, whereas systems with lower Spc values exhibited comparatively narrow confidence intervals, suggesting more homogeneous surface peak distributions.

**Figure 10:**
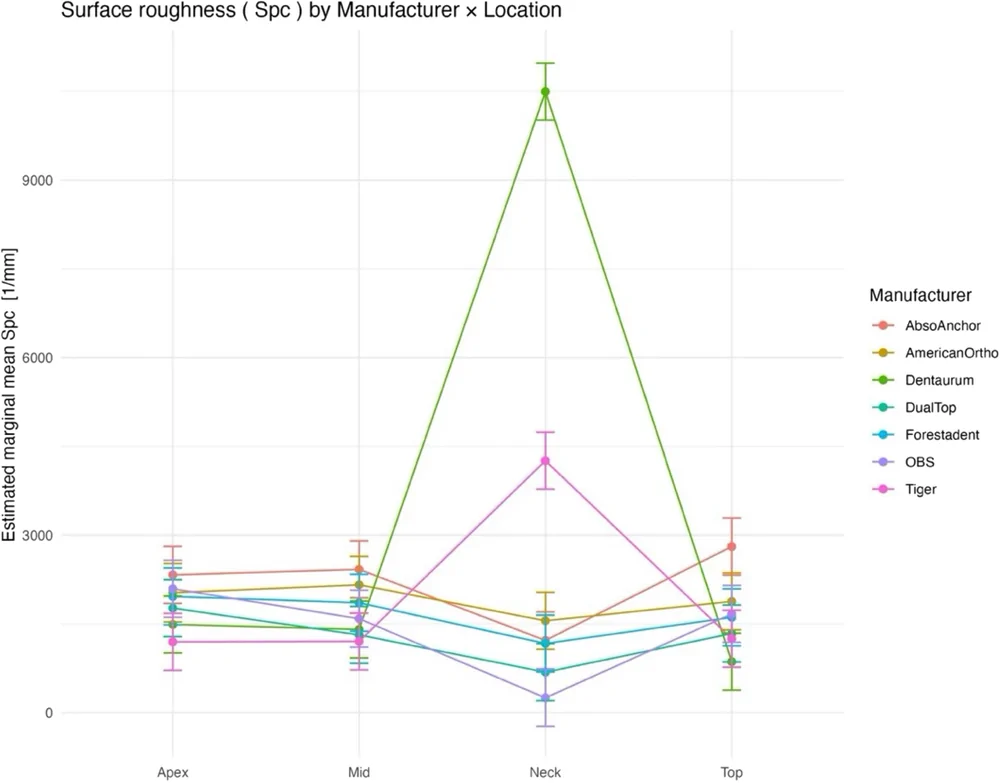
Estimated marginal means (±95% CI) of Spc values (1/mm) for each manufacturer across measurement locations.

Post hoc pairwise comparisons revealed large to extremely large standardized differences between specific manufacturer–location combinations, with standardized mean differences ranging from approximately *d* ≈ 1.6 to > 4.0, confirming pronounced manufacturer‐specific differences in peak density across implant regions.

## 4 Discussion

The present study was designed to systematically compare the surface roughness of different OMIs at multiple positions along the implant body. To our knowledge, such positional analysis has not previously been reported. This information is clinically relevant because surface features in distinct OMI regions may differently affect osseointegration, soft‐tissue healing, and long‐term stability of OMIs.

Across all surface roughness parameters analyzed, the results revealed clear manufacturer‐specific and position‐dependent patterns. In the present study, 3D parameters were preferred over 2D profile parameters due to their ability to provide a comprehensive and detailed representation of surface complexity, a trend increasingly seen in implant dentistry (Fahlstedt et al. 2023; He et al. 2023; Ichioka et al. 2022; Mistretta et al. 2024). Following the suggestions of Wennerberg and Albrektsson in their guidelines for the topographic evaluation of implant surfaces, investigation was not limited to height parameters (Sa, Sz), but also to spacial (Str), hybrid (Sdr), as well as feature (Spc) parameters to better assess implant surface features (Wennerberg and Albrektsson 2000).

Dentaurum consistently exhibited the most pronounced roughness values, particularly at the neck region, where Sa, Sz, Sdr, and Spc all reached levels far exceeding those of the other systems. Ortholox Plus also showed elevated roughness and height values at the neck, although to a lesser extent, while OBS demonstrated the opposite trend with comparatively smooth surfaces, reflected in low Sa, Sz, Sdr, and Spc values. AbsoAnchor, AmericanOrtho, DualTop, and Forestadent displayed moderate profiles overall, with fewer site‐specific variations. Importantly, the neck emerged as the region where roughness differences between systems were most accentuated, whereas mid and apex locations tended to be more consistent across manufacturers.

These manufacturer‐specific differences may also be attributable to variations in the manufacturing and post‐processing steps (Ercetin et al. 2023).

These findings have important implications for the biological response and clinical performance of OMIs. Moderate roughness at the OMI screw thread enhances primary stability by increasing bone‐to‐implant contact and mechanical retention, crucial for immediate or early orthodontic loading. Immediate loading is applied when performing a one‐visit protocol, where the OMIs and the orthodontic appliance are placed in a single appointment (Augustinowitz et al. 2026). Animal studies have shown that immediate loading with light orthodontic forces of 50 cN can be histologically well‐tolerated, without impairing peri‐implant bone tissue reaction (Luzi et al. 2009). Immediate loading is also associated with secondary stability through ongoing bone remodeling around the OMIs (Figueiredo et al. 2024; Selvaraj et al. 2024).

Conversely, smoother surfaces at the transmucosal neck, especially observed in OBS OMIs, reduce plaque retention and inflammation risks. The mostly low Sa values suggest that manufacturers typically engineer the transmucosal neck to be smoother, likely to minimize soft tissue irritation and plaque accumulation (Guo et al. 2021; Quirynen et al. 1993; Teughels et al. 2006). Nevertheless, the differences observed at the neck region suggest that manufacturers should prioritize achieving a smoother neck surface to minimize biofilm accumulation and support peri‐implant soft‐tissue health.

The marked anisotropy (low Str) of several systems suggests pronounced directional texturing and the presence of an engineered surface, which may influence not only osseointegration but also mechanical fatigue resistance. By contrast, the more isotropic surfaces of AmericanOrtho and Forestadent OMIs indicate more uniform texturing, which could offer advantages in terms of reproducibility and long‐term stability.

Although the investigated OMIs have been in clinical use for several years, no systematic comparison of their surface roughness has yet been conducted. In contrast, similar analyses are commonly performed in dental implantology, providing valuable insights. Multiple studies have demonstrated that moderately rough surfaces (Sa ≈ 1–2 µm) promote osseointegration, whereas surfaces that are either too smooth or excessively rough tend to be less favorable (Buser et al. 1991; Wennerberg and Albrektsson 2010). The available data for OMIs is limited and often restricted to single products or global comparisons between manufacturers (Asscherickx et al. 2005; Costa et al. 1998; Motoyoshi et al. 2010). The present study extends current knowledge by demonstrating that surface roughness is not uniform along the implant body and that neck characteristics differ substantially between systems. In particular, the extreme roughness increase observed in Dentaurum OMIs at the neck has not previously been documented and may affect the biological response. Conversely, the consistently smooth neck surface of OBS OMIs aligns with previous work highlighting reduced bacterial colonization on smoother transmucosal regions (Quirynen et al. 1993; Teughels et al. 2006). These findings suggest that design strategies well established in dental implants—balancing roughness for osseointegration with smoothness for biofilm control—are equally relevant in OMIs, yet current systems show strikingly different approaches to this balance. Importantly, these differences may have clinical implications, particularly in regions such as the alveolar ridge, where OMI survival rates remain considerably lower than in the anterior palate (Hourfar et al. 2017). As biofilm accumulation is more likely in these areas, surface characteristics, especially of the transmucosal neck, may play a role in long‐term success, since gingival inflammation has been associated with OMI failure. Nevertheless, the main contributing factor of OMI failure is inadequate primary mechanical stability (Chen et al. 2009; Vicioni‐Marques et al. 2022).

The findings of this study should be interpreted in light of certain limitations. First, the analyses were conducted on a limited number of OMIs per manufacturer, which may not capture the full variability of production batches. Moreover, the large number of parameters, locations, and pairwise comparisons evaluated relative to the sample size represents a considerable analytical burden. Although a post hoc sensitivity analysis was performed for Sa, it indicated that the present sample size was mainly able to detect moderate‐to‐large between‐manufacturer differences; therefore, smaller location‐specific differences may have remained undetected. Second, the study design was entirely in vitro, focusing on topographical parameters derived from confocal microscopy. While these measures are highly relevant to surface characterization, they do not directly translate into biological outcomes such as osseointegration or soft‐tissue integration. Third, only selected roughness parameters (Sa, Sz, Sdr, Str, Spc) were analyzed; although these provide a comprehensive view of topography, other parameters, including analyses of OMI macro topography, could yield additional insights. More specifically, thread design itself influences stress concentration and distribution at the bone‐implant interface, an aspect not evaluated in the present study but relevant to mechanical performance. Finite element analyses have shown that also implant diameter and insertion angle considerably affect stress distribution in the OMI and surrounding bone during orthodontic loading, with narrower OMIs and increased insertion angles associated with higher stress concentrations (Sivamurthy and Sundari 2016). Finally, no longitudinal biological validation was performed in vivo, and therefore, clinical implications regarding stability or peri‐implant health remain hypothetical. Future research should combine surface topography with biomechanical testing and biological assays to establish clearer links between roughness at specific OMI locations and clinical performance.

Future studies should extend these findings by correlating detailed surface roughness measurements with biological and clinical outcomes. In particular, longitudinal in vivo studies are needed to evaluate how neck versus mid or apex surface characteristics influence initial stability, peri‐implant bone remodeling, and soft‐tissue health. Integration of microbiological analyses could clarify the role of surface topography in bacterial adhesion and biofilm development, while advanced biomechanical testing may provide insights into fatigue resistance and fracture risk. Ultimately, linking positional roughness characteristics of OMIs to patient‐centered outcomes will be essential for guiding optimized design strategies that balance osseointegration, stability, and peri‐implant tissue health.

## 5 Conclusion

This study systematically compared surface roughness parameters of OMIs across multiple manufacturers and implant regions. The results demonstrated pronounced manufacturer‐specific and position‐dependent differences, with Dentaurum showing the highest roughness values, particularly at the neck, while OBS consistently exhibited the smoothest profiles. These findings highlight that surface characteristics are not uniform along the implant body and may carry important implications for osseointegration, peri‐implant tissue health, and long‐term stability. Understanding the influence of these surface features in clinical settings could lead to the selection of specific implants tailored to particular patient needs or even the customization of implant surfaces to optimize clinical outcomes. The present data provide a foundation for further translational and clinical studies aimed at optimizing OMI surface design.

## Author Contributions

**Kathrin Becker** and **Alessandro Antonelli:** conceptualization. **Till Augustinowitz, Kathrin Becker,** and **Alessandro Antonelli:** methodology. **Kathrin Becker** and **Alessandro Antonelli:** software. **Till Augustinowitz, Kathrin Becker, Giulia Brunello,** and **Alessandro Antonelli:** formal analysis. **Alessandro Antonelli:** investigation. **Kathrin Becker:** resources. **Till Augustinowitz, Kathrin Becker,** and **Alessandro Antonelli:** data curation. **Till Augustinowitz** and **Kathrin Becker:** writing – original draft preparation. **Till Augustinowitz, Giulia Brunello, Kathrin Becker,** and **Alessandro Antonelli:** writing – review and editing. **Till Augustinowitz, Kathrin Becker,** and **Alessandro Antonelli:** visualization. **Kathrin Becker:** supervision. **Kathrin Becker:** project administration. **Kathrin Becker:** funding acquisition.

## Funding

The authors have nothing to report.

## Conflicts of Interest

The authors declare no conflicts of interest.

## Supporting information


Supporting File 1

Supporting File 2

## Data Availability

Data will be provided upon reasonable request.
