# Supplementary material for: Evaluation of Positional Variability in Surface Roughness in Orthodontic Mini‐Implants Using Confocal Microscopy: A Multi‐Manufacturer Comparative Study
Source: Clin Exp Dent Res. 2026 Oct 29;12(5):e70497. doi: 10.1002/cre2.70497 (PMC13719047; doi:10.1002/cre2.70497)
Supplement: Supplementary file 1 [file CRE2-12-e70497-s001.docx]

**Supplementary Figures**

**Suppl. Figure 1.** Illustration of laser-based optical surface roughness measurements at the Top for all seven manufacturers with related rendering of the analysed digital optical impression (200 μm × 200 μm), created with MultiFileAnalyzer software (KEYENCE, Osaka, Japan).

**Suppl. Figure 2.** Illustration of laser-based optical surface roughness measurements at the Mid for all seven manufacturers with related rendering of the analysed digital optical impression (200 μm × 200 μm), created with MultiFileAnalyzer software (KEYENCE, Osaka, Japan).

**Suppl. Figure 3.** Illustration of laser-based optical surface roughness measurements at the Apex for all seven manufacturers with related rendering of the analysed digital optical impression (200 μm × 200 μm), created with MultiFileAnalyzer software (KEYENCE, Osaka, Japan).
